# An improved secretion signal enhances the secretion of model proteins from *Pichia pastoris*

**DOI:** 10.1186/s12934-018-1009-5

**Published:** 2018-10-12

**Authors:** Juan J. Barrero, Jason C. Casler, Francisco Valero, Pau Ferrer, Benjamin S. Glick

**Affiliations:** 1 Department of Chemical, Biological and Environmental Engineering, Escola d’Enginyeria, Universitat Autònoma de Barcelona, Bellaterra (Cerdanyola del Vallès), 08193 Catalonia, Spain; 2 Department of Molecular Genetics and Cell Biology, University of Chicago, 920 East 58th Street, Chicago, IL 60637 USA; 3 Present Address: Luxembourg Institute of Science and Technology (LIST), Belvaux, Luxembourg

**Keywords:** Translocation, Secretion, *Pichia pastoris*, Alpha-factor, Ost1, Heterologous protein production, Aggregation

## Abstract

**Background:**

Proteins can be secreted from a host organism with the aid of N-terminal secretion signals. The budding yeast *Pichia pastoris* (*Komagataella* sp.) is widely employed to secrete proteins of academic and industrial interest. For this yeast, the most commonly used secretion signal is the N-terminal portion of pre-pro-α-factor from *Saccharomyces cerevisiae*. However, this secretion signal promotes posttranslational translocation into the endoplasmic reticulum (ER), so proteins that can fold in the cytosol may be inefficiently translocated and thus poorly secreted. In addition, if a protein self-associates, the α-factor pro region can potentially cause aggregation, thereby hampering export from the ER. This study addresses both limitations of the pre-pro-α-factor secretion signal.

**Results:**

We engineered a hybrid secretion signal consisting of the *S. cerevisiae* Ost1 signal sequence, which promotes cotranslational translocation into the ER, followed by the α-factor pro region. Secretion and intracellular localization were assessed using as a model protein the tetrameric red fluorescent protein E2-Crimson. When paired with the α-factor pro region, the Ost1 signal sequence yielded much more efficient secretion than the α-factor signal sequence. Moreover, an allelic variant of the α-factor pro region reduced aggregation of the E2-Crimson construct in the ER. The resulting improved secretion signal enhanced secretion of E2-Crimson up to 20-fold compared to the levels obtained with the original α-factor secretion signal. Similar findings were obtained with the lipase BTL2, which exhibited 10-fold enhanced secretion with the improved secretion signal.

**Conclusions:**

The improved secretion signal confers dramatic benefits for the secretion of certain proteins from *P. pastoris*. These benefits are likely to be most evident for proteins that can fold in the cytosol and for oligomeric proteins.

**Electronic supplementary material:**

The online version of this article (10.1186/s12934-018-1009-5) contains supplementary material, which is available to authorized users.

## Background

In recent decades, the methylotrophic yeast *Pichia pastoris* (*Komagataella* sp.) has become one of the most popular platforms for heterologous protein production [1–4]. This popularity stems from several advantages of *P. pastoris*, including its capacity to grow in both defined and complex media, the possibility to reach high cell densities of up to 130 g/L of dry cell weight [5], and availability of the strong and tightly regulatable *AOX1* (alcohol oxidase 1) promoter, which can be induced with methanol or repressed with glucose or glycerol [6]. Furthermore*, P. pastoris* secretes only low levels of endogenous proteins, a property that facilitates downstream processing because the heterologous protein comprises the vast majority of the protein in the medium. *P. pastoris* is designated a Generally Recognized As Safe (GRAS) organism, and its similarity to *Saccharomyces cerevisiae* enables the sharing of protocols and of certain genetic elements such as secretion signals.

For heterologous protein production in *P. pastoris*, the most common secretion signal is that of the *S. cerevisiae* α-factor mating pheromone [7]. This secretion signal consists of two parts: a 19-amino acid N-terminal signal sequence that directs translocation into the endoplasmic reticulum (ER), followed by a 66-amino acid pro region that mediates receptor-dependent packaging into ER-derived COPII transport vesicles (Additional file 1: Figure S1) [8, 9]. The α-factor signal sequence is removed by a signal peptidase in the ER lumen, and the pro region is cleaved by the Kex2 processing protease in the Golgi [10, 11]. This bipartite secretion signal has proven to be effective for secreting multiple heterologous proteins in *P. pastoris*, but the level of secretion varies widely, prompting efforts to improve secretion efficiency [4, 12–16]. Several of those attempts focused on modifying the α-factor secretion signal [17–20].

A limitation of the α-factor secretion signal is that the signal sequence portion directs posttranslational translocation across the ER membrane [21, 22]. As a result, if the α-factor secretion signal is fused to a protein that can fold in the yeast cytosol, the protein may be unable to cross the ER membrane and enter the secretory pathway. We encountered this problem when monitoring secretion in *S. cerevisiae* or *P. pastoris* using a monomeric superfolder GFP (msGFP) [23]. The solution was to replace the α-factor signal sequence with the Ost1 signal sequence, which directs cotranslational translocation across the ER membrane, thereby ensuring that msGFP folds only after reaching the ER lumen [24, 25].

Here, we have extended this analysis by using the tetrameric far-red fluorescent protein E2-Crimson as a model for heterologous protein secretion in *P. pastoris*. E2-Crimson folds and oligomerizes efficiently, and it acquires fluorescence rapidly [26]. The fluorescence signal provides a convenient way to visualize potential roadblocks in the secretory pathway using fluorescence microscopy. There are advantages to using E2-Crimson instead of further investigating secretion of msGFP. E2-Crimson is oligomeric, so the data are complementary to those obtained with the monomeric msGFP. Moreover, E2-Crimson fluoresces at red wavelengths, so we can track secretion without interference from the green fluorescence of riboflavin, a yeast culture medium component that is produced at high levels by methanol-grown *P. pastoris* cells [27, 28].

Our results indicate that E2-Crimson can become trapped along the secretory pathway in two ways. First, when the α-factor signal sequence is used, E2-Crimson fails to cross the ER membrane, presumably because the protein folds prior to posttranslational translocation. As with msGFP, this problem can be overcome by using the Ost1 signal sequence. Second, when fused to a commonly used variant of the α-factor pro region, E2-Crimson aggregates in the ER lumen, presumably because the pro region has a self-association tendency that is amplified by the oligomeric nature of E2-Crimson. This problem can be overcome with an allelic variant in which a single amino acid difference in the pro region suppresses aggregation. Combining the two modifications yielded an improved secretion signal that drives highly efficient secretion of E2-Crimson.

An important question is whether these improvements extend beyond the model fluorescent proteins. As a case study, we chose the BTL2 lipase from *Bacillus thermocatenulatus* [29, 30]. Lipases are of major industrial value [31], and BTL2 is promising because it is thermostable as well as catalytically active at high pH and in the presence of organic solvents [29, 32]. *P. pastori*s has been engineered to secrete BTL2 [33]. A recent study of BTL2 secretion from *S. cerevisiae* showed that the choice of secretion signal was particularly important [34], hinting that BTL2 might be prone to folding prior to translocation. In support of this idea, use of the improved secretion signal in *P. pastoris* strongly enhances BTL2 secretion. This finding suggests that the improved secretion signal will be broadly useful.

## Methods

### Strains and plasmids

*Pichia pastoris* strains were derivatives of X-33 (Thermo Fisher Invitrogen). All strains were selected and grown in rich medium (YPD) supplemented with either Zeocin (100 µg/mL), hygromycin (250 µg/mL), or G418 (500 µg/mL) depending on the integrated plasmid. Buffered minimal glycerol (BMG) and buffered minimal methanol (BMM) media recipes were taken from the instruction manual for Thermo Fisher Invitrogen’s *Pichia* Expression Kit.

Plasmids were created and modified by standard methods including site-directed mutagenesis [35] and In-Fusion cloning (TaKaRa/Clontech). Primers were purchased from IDT. The gene encoding BTL2 was codon-optimized for *P. pastoris* by GenScript. Expression of E2-Crimson and BTL2 were driven by the inducible *AOX1* promoter, while expression of msGFP-HDEL and Htb2-GFP were driven by the *KAR2* and *GAP* promoters, respectively. Genetic engineering procedures were designed and recorded using SnapGene software (GSL Biotech). The supplementary information contains a compressed folder of SnapGene files for the plasmids used in this study (Additional file 2), and those files can be opened with the free SnapGene Viewer (http://www.snapgene.com/products/snapgene_viewer). Key plasmids will be deposited with Addgene.

Plasmids were linearized and then transformed by electroporation [36] using 100 ng of linear DNA, an amount that limited the number of copies integrated. Single-copy integration was confirmed by PCR of purified genomic DNA using primers that flanked the integration locus. The E2-Crimson and BTL2 constructs were integrated at the *AOX1* locus, and single-copy integration was verified using primers 5′-GAAATAGACGCAGATCGGGAAC-3′ and 5′-GAAGGTAGACCCATGGGTTGTTG-3′. The pre-Kar2-msGFP-HDEL construct was integrated at the *HIS4* locus, and single-copy integration was verified using primers 5′-GCTCTAGCCAGTTTGCTGTCCAAAC-3′ and 5′-GGATGTTAGATGCCGGTTAGATC-3′. The Htb2-GFP construct was integrated at the *GAP* locus, and single-copy integration was verified using primers 5′-GATGACAATGGACCAAATTGTTGCAAGG-3′ and 5′-CCGTTAATACCGACAGTGATAGCC-3′. Additionally, for strains with BTL2 constructs, droplet digital PCR [37] was performed to confirm single-copy integration using primers 5′-GGGTATGAACGCTTTTTCTGCTGTTG-3′ and 5′-GATCAACGTTACAAGTACCCATATCATTCC-3′ for the BTL2 gene, or 5′-CCTGAGGCTTTGTTCCACCCATCT-3′ and 5′-GGAACATAGTAGTACCACCGGACATAACGA-3′ for the actin gene as a control.

### Assaying secretion of E2-Crimson

From each strain, eight transformed colonies were streaked on YPD plates containing the appropriate antibiotic and then re-streaked twice on new plates to avoid mixed cell populations. After identifying single-copy integrants for each strain, a pre-screening of six clones to assess the level of E2-Crimson secretion (Additional file 1: Figure S2) was used to identify two representative clones. Further analysis was performed in parallel with those two clones, which were stored frozen at − 80 °C. Clones were retrieved from the frozen stocks to make saturated YPD precultures that were kept for up to 2–3 weeks at 4 °C.

For a given strain, each of the two clones was analyzed in triplicate as follows. The E2-Crimson secretion assay was initiated by inoculating a 1:1000 dilution of a preculture into 5 mL of YPD in a 15-mL culture tube. After a day of incubation at 30 °C with shaking at 220 rpm in an Infors HT incubator, an aliquot of the culture was diluted to an optical density at 600 nm (OD_600_) of 0.2 in 5 mL of BMG in a 15-mL culture tube. This tube was incubated under the same conditions as before. The following day, the culture was centrifuged at 3000 rpm (2000×*g*) for 5 min and resuspended in 25 mL BMM to attain an OD_600_ of 1.0. 25 mL of this culture was placed in a 250-mL baffled flask (Corning), and during this induction phase, the cells were incubated at 25 °C with shaking at 150 rpm to reduce loss of methanol. Additional flasks containing water were present in the shaker to generate a humid atmosphere and minimize evaporation. After 1 day of induction, an additional dose of 125 µL methanol was added (yielding a final concentration of 0.5%), and the incubation was continued for another day. After 48 h of induction, 1.5 mL of the culture was centrifuged at 6000 rpm (2000×*g*) in a microcentrifuge for 2 min to separate the cell pellet from the supernatant. The cell pellet was washed with phosphate buffered saline (PBS) and then resuspended in 1.5 mL PBS.

300 µL each of the pellet and supernatant fractions were transferred in triplicate to a 96-well plate (Costar) to measure E2-Crimson fluorescence using a Synergy Neo Microplate Reader (BioTek). The excitation and emission wavelengths were 611 nm and 646 nm, respectively, and the gain was 150. The results were normalized by dividing by the final OD_600_ value for the culture.

### Assaying secretion of BTL2

The basic procedures described above for E2-Crimson were adapted to obtain strains expressing BTL2 and to monitor BTL2 secretion. To quantify the secretion of BTL2, lipolytic activity was measured in duplicate using a lipase colorimetric assay (Roche Diagnostics). Briefly, 0.5 mL of a suitably diluted supernatant from each strain was mixed with 0.5 mL Tris–HCl buffer (200 mM, pH 7.25), placed in a thermostatically controlled cuvette, and incubated at 50 °C for 5 min. Then 0.3 mL of substrate (1,2-*O*-dilauryl-rac-glycero-3-glutaric-(methylresorufin)-ester) was mixed with the pre-warmed sample and monitored at 580 nm for 7 min in a Specord 200 Plus Spectrophotometer (Analytic Jena). Lipolytic activity was measured using the values between minutes 3 and 5. The absorbance increase per second was used to determine the protein activity, with one unit of lipolytic activity defined as the amount of lipase needed to hydrolyze 1 μmol of ester bond per minute.

### Immunoblot for E2-Crimson

Strains expressing E2-Crimson were grown and induced as described above. A 25-mL culture was centrifuged at 4 °C at 4700 rpm (5000×*g*) for 5 min, then the supernatant was carefully removed and placed on ice. 111 µL of 100% w/v trichloroacetic acid (TCA) was added per mL of supernatant. The sample was vortexed briefly and left on ice for 20 min. Then the sample was centrifuged at maximum speed in a microcentrifuge for 15 min. The supernatant was discarded, and the precipitate was washed with 100% ethanol, resuspended in 50 µL SDS-PAGE sample buffer, boiled for 10 min, vortexed, and centrifuged at maximum speed in a microcentrifuge for 5 min to remove insoluble material. A 20-µL aliquot was loaded on a 4–20% Mini-PROTEAN TGX Precast Protein Gel (Bio-Rad).

Meanwhile, the cell pellet was washed twice with 5 mL H_2_O and then resuspended in 5 mL H_2_O. A 1-mL aliquot was transferred to a snap-cap tube. The cells were centrifuged at 5000 rpm (1400×*g*) in a microcentrifuge for 5 min. The supernatant was discarded, and the pellet was resuspended in 250 µL 20% w/v TCA. Then 250 µL of 0.5-mm glass beads were added and the sample was vortexed at maximum speed for three 1-min pulses, with 1 min on ice between pulses. 800 µL of 5% (w/v) TCA was added, the sample was briefly mixed, and 800 µL of the liquid was transferred to a fresh snap-cap tube and left on ice for 15 min. Finally, the sample was centrifuged and processed in the same manner as the sample from the supernatant containing the secreted proteins, except that the sample from the cell pellet was resuspended in 100 µL SDS-PAGE sample buffer.

Proteins were transferred from the SDS-PAGE gel to a PVDF membrane using the Trans-Blot Turbo System (Bio-Rad). The membrane was then blocked for 1 h with shaking at room temperature in TBST + 5% milk, where TBST is TBS (50 mM Tris–HCl at pH 7.6, 150 mM NaCl) plus 0.05% Tween 20. The blocked membrane was incubated with shaking overnight at 4 °C in TBST + 5% milk containing a 1:500 dilution of Living Colors DsRed Monoclonal Antibody (Clontech/TaKaRa). Then the membrane was washed three times for 5 min each in TBST, and incubated with TBST + 5% milk containing a 1:1000 dilution of goat anti-mouse antibody conjugated to Alexa Fluor 647 (Thermo Fisher) for 1 h. After three washes in TBST, the membrane was washed once more in TBS prior to imaging with a LI-COR Odyssey CLx imaging system.

### Fluorescence microscopy

Images for Fig. 3 and Additional file 1: Figure S4 were captured after the 48-h induction period. Prior to imaging, cultures were spun briefly in a microcentrifuge and then resuspended in PBS to avoid fluorescence background from secreted E2-Crimson.

For Figs. 4 and 7, the strains were grown in BMG and then transferred to a 5-mL culture tube containing BMM for induction at a starting OD_600_ of 0.2. The following day, the cultures were processed and imaged as in Fig. 3.

Images for Fig. 3 and Additional file 1: Figures S4, S5 were captured as Z-stacks using an LSM 880 confocal microscope (Zeiss) equipped with a 1.4-NA/100× oil objective. Images for Fig. 7 were captured as Z-stacks using an SP5 confocal microscope (Leica) equipped with a 1.4-NA/63× oil objective. The Z-stacks were average projected, and the brightness and contrast were adjusted evenly in all images. A Gaussian blur filter was used to smooth the red and green signals. Image processing was performed using ImageJ (https://imagej.nih.gov/ij/).

### Box and whisker plots

These plots were generated with GraphPad Prism. Each box extends from the 25th to 75th percentiles, with the internal line representing the median. Individual data points are shown as dots, and the whiskers mark the minimum and maximum values.

## Results

### The Ost1 signal sequence and a variant of the α-factor pro region synergistically promote efficient secretion of E2-Crimson

**Fig. 1:**
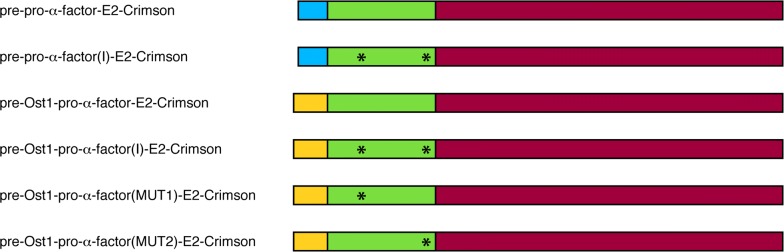
Constructs used in this study. Blue is the α-factor signal sequence, green is the α-factor pro region, yellow is the Ost1 signal sequence, and red is E2-Crimson. The wild-type α-factor pro region variant designated here as pro-α-factor contains Leu42. The pro-α-factor(I) or “Invitrogen” variant contains Ser42 as well as the Asp83-to-Glu mutation (see Additional file 1: Figure S1), as represented by the asterisks. The “MUT1″ and “MUT2” variants of the α-factor pro region contain individual Ser42 and Glu83 mutations, respectively, relative to the Leu42 variant

It was previously shown that the Ost1 signal sequence is more effective than the α-factor signal sequence at promoting secretion of msGFP [23]. Our goal was to test whether those findings could be extended to an oligomeric model protein. For this purpose, we generated a pre-pro-α-factor-E2-Crimson construct, which contains the α-factor signal sequence and pro region, and compared it to a pre-Ost1-pro-α-factor-E2-Crimson construct, which contains the Ost1 signal sequence and the α-factor pro region (Fig. 1).

The other variable we tested was the sequence of the α-factor pro region. Our earlier work employed a pro region variant that contains Leu at position 42 (where the numbering is based on the pre-pro-α-factor precursor) (Additional file 1: Figure S1). This Leu42 variant is commonly used for both biotechnology and basic science applications [8, 38]. By contrast, the originally described allele of the α-factor gene contains Ser at position 42 [39]. This Ser42 variant is present in Invitrogen’s widely used pPICZα family of plasmids, which also contain a trio of point mutations that create an *Xho*I restriction site while changing Asp83 to Glu. The Leu42 variant of the pro region is referred to here simply as pro-α-factor, while the “Invitrogen” Ser42 variant with the XhoI site is referred to as pro-α-factor(I). The constructs with the α-factor and Ost1 signal sequences were modified to include pro-α-factor(I), yielding a total of four constructs that represented all combinations of the signal sequences and pro regions (Fig. 1).

**Fig. 2:**
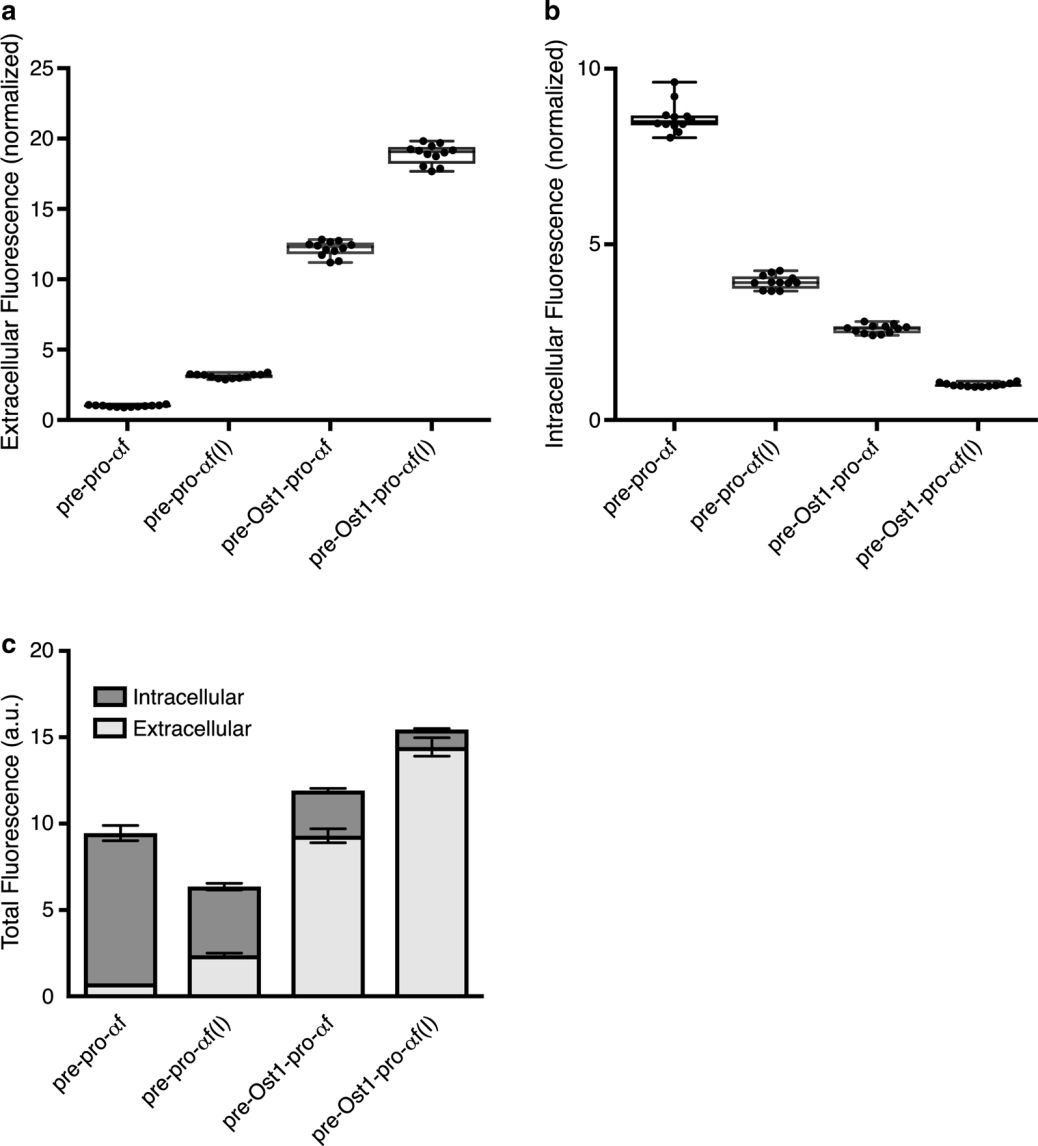
Extracellular and intracellular fluorescence signals with the different secretion signals. Fluorescence signals for extracellular and intracellular E2-Crimson were measured by fluorimetry after 48 h of methanol induction using different secretion signals. Pre-pro-αf, α-factor signal sequence followed by pro-α-factor; pre-pro-αf(I), α-factor signal sequence followed by pro-α-factor(I); pre-Ost1-pro-αf, Ost1 signal sequence followed by pro-α-factor; pre-Ost1-pro-αf(I), Ost1 signal sequence followed by pro-α-factor(I). **a** E2-Crimson fluorescence in the culture medium for the different secretion signals. Each fluorescence signal was divided by the OD_600_ at the end of the incubation. Then the signals were normalized by setting the signal for pre-pro-αf to 1. **b** Same as **a**, except that intracellular fluorescence signals were normalized by setting the signal for pre-Ost1-pro-αf(I) to 1. **c** Total extracellular and intracellular signals are plotted for the different secretion signals. *a.u.* arbitrary units

Expression of these constructs was driven by the *AOX1* promoter [6]. After 48 h of methanol induction, the levels of intracellular and extracellular E2-Crimson fluorescence were measured using a fluorimeter. For each construct, six single-copy integrant clones were tested to confirm that the results were reasonably consistent (Additional file 1: Figure S2), and two representative clones were used for further analysis. The pre-pro-α-factor-E2-Crimson reference construct contained the α-factor signal sequence followed by pro-α-factor. Figure 2a shows that in the context of the α-factor signal sequence, pro-α-factor(I) increased secretion ~ 3-fold. In a parallel test, when paired with pro-α-factor, the Ost1 signal sequence increased secretion ~ 12-fold. When the Ost1 signal sequence was paired with pro-α-factor(I), secretion was increased up to 20-fold (Fig. 2a). The amount of intracellular fluorescence (Fig. 2b) was inversely correlated with the amount of extracellular fluorescence (Fig. 2a). Total fluorescence recovery was highest with the pre-Ost1-pro-α-factor(I) construct (Fig. 2c), possibly because the other constructs led to degradation of protein molecules that failed to be secreted. These results indicate that an improved secretion signal consisting of the Ost1 signal sequence and pro-α-factor(I) is remarkably effective at promoting secretion of E2-Crimson.

The fluorescence measurements were verified qualitatively by immunoblotting (Additional file 1: Figure S3). A protein that migrated at the position expected for mature E2-Crimson (26 kDa) was seen in the medium, and this gel band was most intense with the pre-Ost1-pro-α-factor(I) construct. Thus, the improved secretion signal appears to be proteolytically processed by *P. pastoris* cells in the same manner as the α-factor secretion signal. Compared to secreted E2-Crimson, cell-associated E2-Crimson migrated more slowly. This gel band was most intense with the constructs containing the α-factor signal sequence. As described below, most of the cell-associated E2-Crimson was probably molecules that failed to cross the ER membrane completely, so the higher apparent molecular weight reflects the presence of an unprocessed or incompletely processed secretion signal.

### Intracellular E2-Crimson constructs become trapped during or after translocation into the ER

**Fig. 3:**
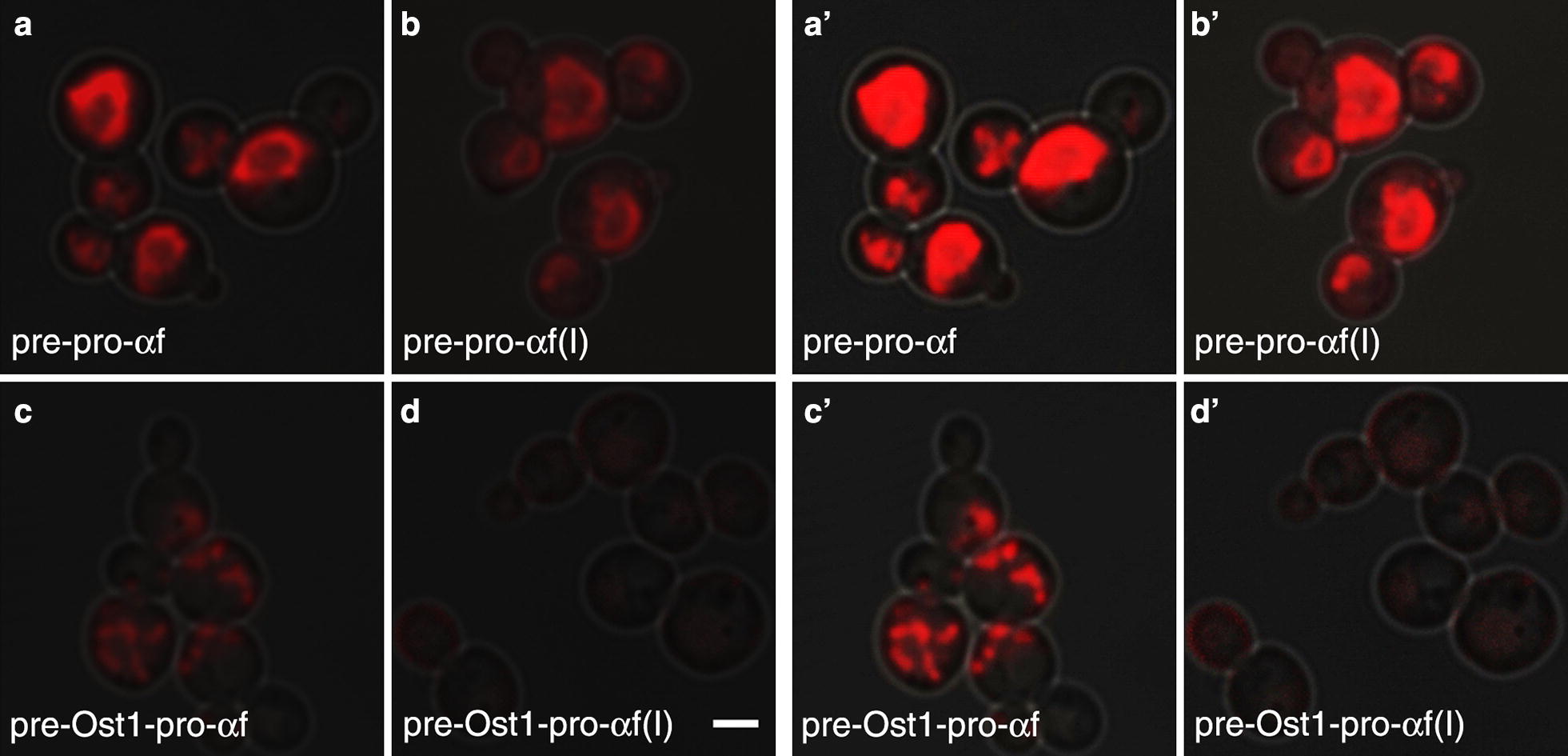
Images of intracellular fluorescence with the different secretion signals. The abbreviations are as in Fig. 2. Projected confocal Z-stacks of E2-Crimson fluorescence were merged with differential interference contrast images of the cells. The rings in **a**, **b** represent the nuclear envelope, and the spots in **c** represent aggregates in the ER lumen. **a**′ through **d**′ are the same images as **a** through **d** but adjusted to a higher brightness level. Scale bar, 2 μm

The constructs that showed substantial intracellular accumulation were presumably becoming trapped at early stages in the secretory pathway. To characterize those bottlenecks, we used fluorescence microscopy to visualize the location of the accumulated E2-Crimson. Figure 3 shows the cellular fluorescence patterns, at two brightness levels, for cells expressing the four constructs described above.

**Fig. 4:**
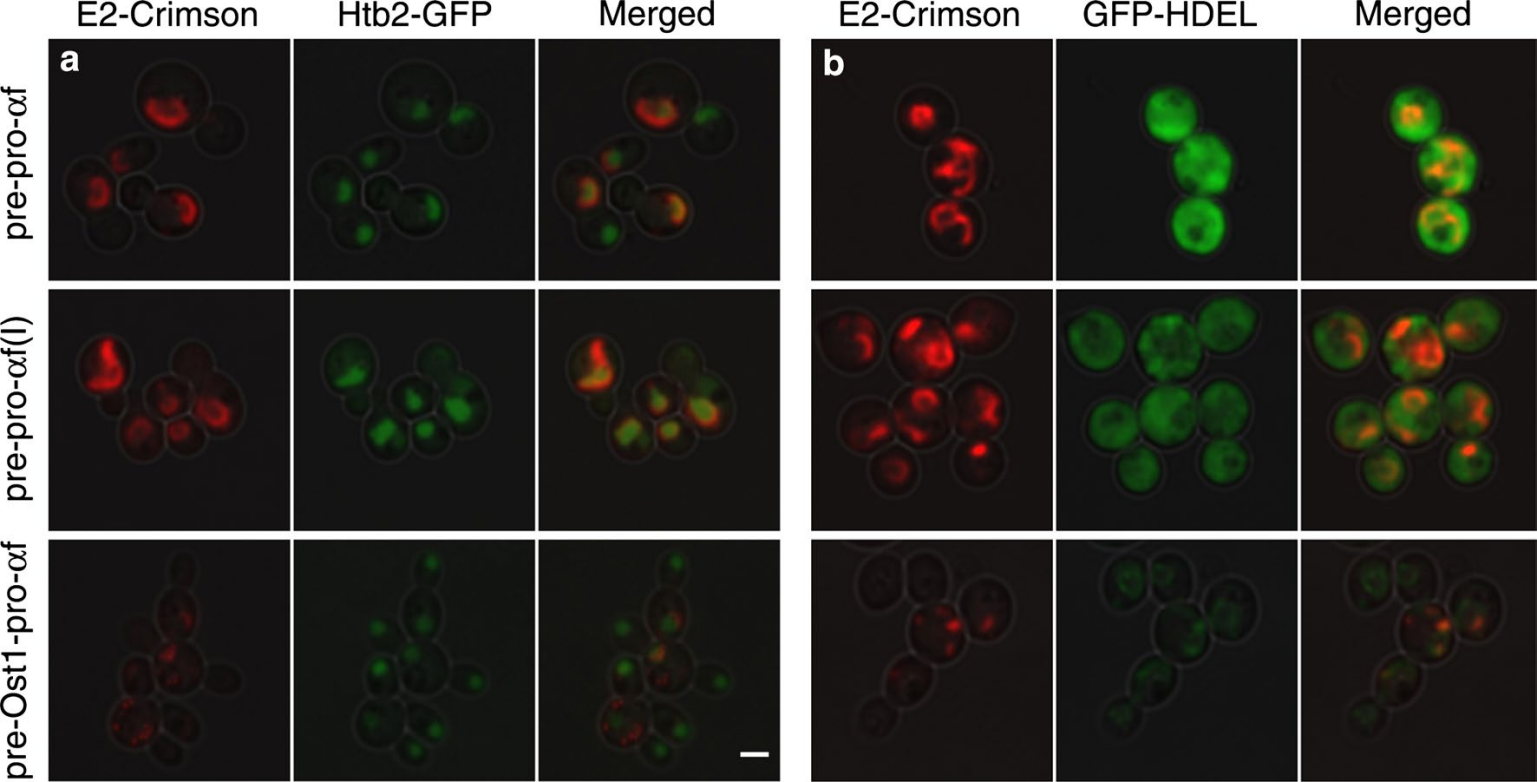
Confirmation that intracellular E2-Crimson constructs were associated with the ER. The abbreviations for the constructs are as in Fig. 2. Projected confocal Z-stacks of the fluorescent proteins were merged with differential interference contrast images of the cells. **a** Htb2-GFP represents histone 2B tagged with GFP to label the nucleus. For the constructs with the α-factor signal sequence, much of the intracellular red fluorescence was in the nuclear envelope. For the construct with the Ost1 signal sequence, punctate aggregates were visible. **b** GFP-HDEL represents ER-targeted GFP with a C-terminal HDEL tetrapeptide for ER retention. For the constructs with the α-factor signal sequence, most of the GFP-HDEL remained in the cytosol. For the construct with the Ost1 signal sequence, GFP-HDEL exhibited a typical ER pattern, and was present in the same locations as the E2-Crimson aggregates. Scale bar, 2 μm

Based on the previous work with msGFP [23], we anticipated that the α-factor signal sequence would drive posttranslational translocation, and would therefore lead to accumulation of translocation intermediates in which the folded E2-Crimson domain remained on the cytosolic side of the ER membrane. Our fluorescence images are consistent with this prediction. The two constructs with the α-factor signal sequence yielded fluorescent rings typical of the ER (Fig. 3a, b), which consists of the nuclear envelope plus peripheral ER elements. Those rings were indeed the nuclear envelope as confirmed by labeling the nuclear DNA with histone H2B (Htb2) fused to GFP (Fig. 4a). For the constructs containing the α-factor signal sequence, their strong ER labeling (Additional file 1: Figure S4) combined with their relatively weak secretion suggests that they were trapped in transit across the ER membrane, with the signal sequences engaging the ER translocation machinery while the folded E2-Crimson domains remained on the cytosolic side of the ER membrane.

If this interpretation is correct, the E2-Crimson constructs containing the α-factor signal sequence might be expected to “clog” the translocons in the ER [40]. To test this hypothesis, we expressed an additional construct in which the Kar2 signal sequence was fused to GFP-HDEL. This fusion protein normally labels the ER lumen [41], and accordingly, we observed rings of ER-localized green fluorescence prior to methanol induction (Additional file 1: Figure S5). However, in methanol-induced cells expressing the E2-Crimson constructs with the α-factor signal sequence, much of the green fluorescence was cytosolic (Fig. 4b). Moreover, cells expressing the E2-Crimson constructs containing the α-factor signal sequence were often unusually large (Additional file 1: Figure S4), consistent with a toxic effect of those constructs. The combined results support the idea that the α-factor signal sequence generates an intermediate that becomes trapped during passage across the ER membrane.

The Ost1 signal sequence drives cotranslational translocation, and should therefore enable E2-Crimson to reach the ER lumen. With the pre-Ost1-pro-α-factor-E2-Crimson construct, there was no labeling of the nuclear envelope. Instead, punctate structures were observed in the cells (Fig. 3c). Those structures were apparently aggregates located in the ER lumen because they also labeled with GFP-HDEL (Fig. 4b). In this strain, the GFP-HDEL was present in the ER rather than the cytosol, indicating that the translocons were not clogged (Fig. 4b). The implication is that the Ost1 signal sequence overcomes the problem of translocating E2-Crimson into the ER but does not prevent subsequent aggregation in the ER lumen.

With the pre-Ost1-pro-α-factor(I)-E2-Crimson construct, in which pro-α-factor was replaced with the “Invitrogen” variant, no aggregates were seen in the ER lumen. Indeed, very little intracellular red fluorescence was seen (Fig. 3d), consistent with the results of the fluorimeter assays (Fig. 2). Our interpretation is that pro-α-factor can lead to aggregation in the ER lumen, and that this effect is avoided by using pro-α-factor(I) instead.

### The superior behavior of pro-α-factor(I) is due to Ser42

We tested whether the enhanced secretion obtained with pro-α-factor(I) was due to one or both of the amino acid differences relative to pro-α-factor. For this purpose, pro-α-factor was modified by introducing either a point mutation that changed Leu42 to Ser, or a point mutation that changed Asp83 to Glu (Additional file 1: Figure S1). These variants were designated pro-α-factor(MUT1) and pro-α-factor(MUT2), respectively (Fig. 1). The MUT1 and MUT2 variants were tested in the context of the Ost1 signal sequence.

**Fig. 5:**
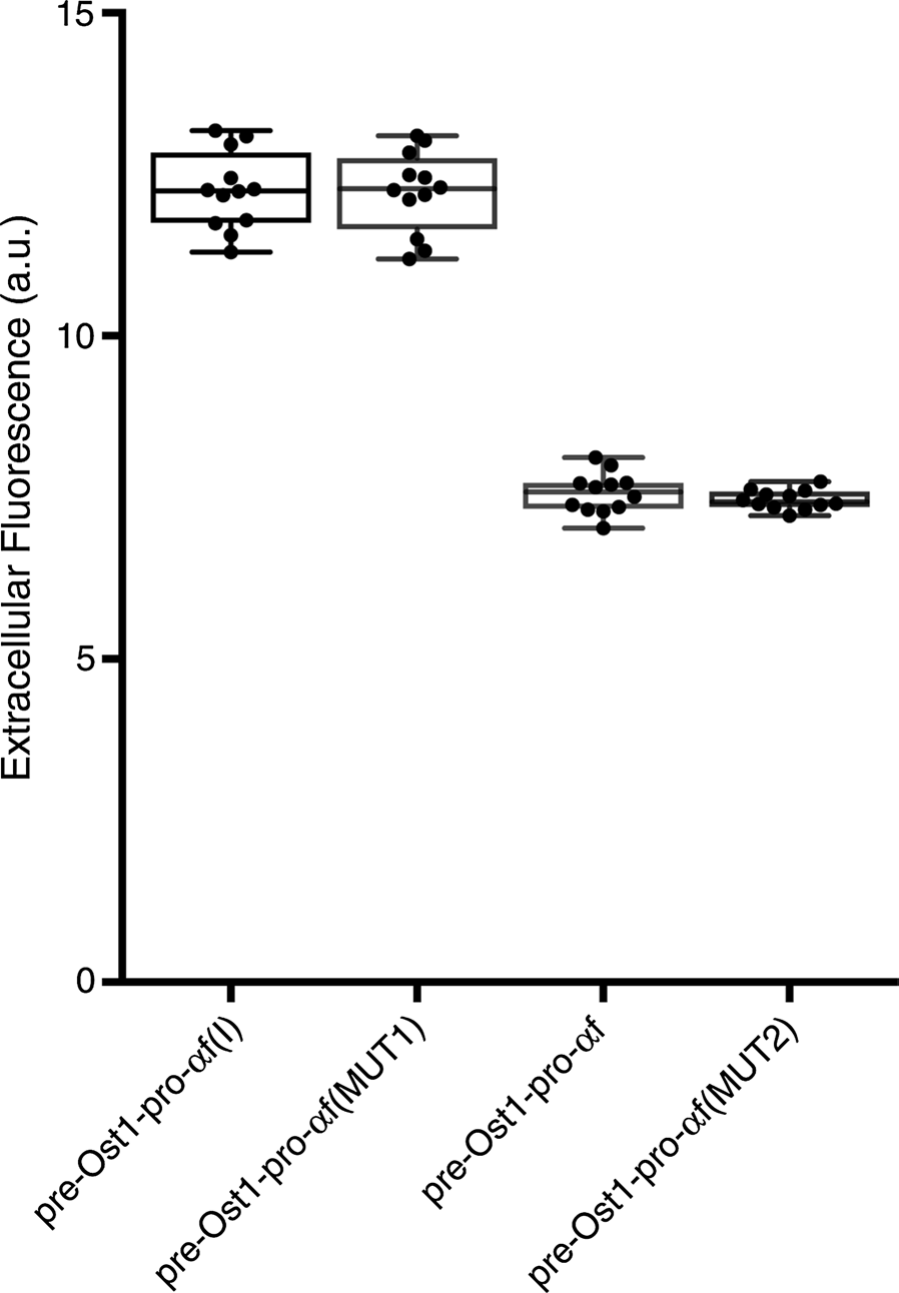
Separate analysis of the two differences that distinguish pro-α-factor(I) from pro-α-factor. The experiment was performed as in Fig. 2a, except that the signals were not normalized and the Ost1 signal sequence was used together with either pro-α-factor, or pro-α-factor(I), or the MUT1 or MUT2 variant of the α-factor pro region. *a.u.* arbitrary units

As shown in Fig. 5, measurements of extracellular fluorescence gave an unambiguous answer: the MUT1 change (Leu42 to Ser) was necessary and sufficient for enhancing E2-Crimson secretion. Moreover, intracellular aggregates were seen in cells expressing pre-Ost1-pro-α-factor(MUT2)-E2-Crimson but not in cells expressing pre-Ost1-pro-α-factor(MUT1)-E2-Crimson (data not shown). To gain insight into the potential mechanism of the MUT1 mutation, we analyzed the α-factor pro region using the program AGGRESCAN, which estimates the aggregation propensity of a polypeptide sequence [42]. A stretch of amino acids containing Leu42 was predicted to be aggregation-prone, and the predicted aggregation propensity was substantially reduced by changing Leu42 to Ser (Additional file 1: Figure S6). We conclude that substitution of Leu42 with the less hydrophobic Ser is crucial for suppressing aggregation of E2-Crimson constructs in the ER.

### Secretion of the BTL2 lipase is enhanced by the improved secretion signal

**Fig. 6:**
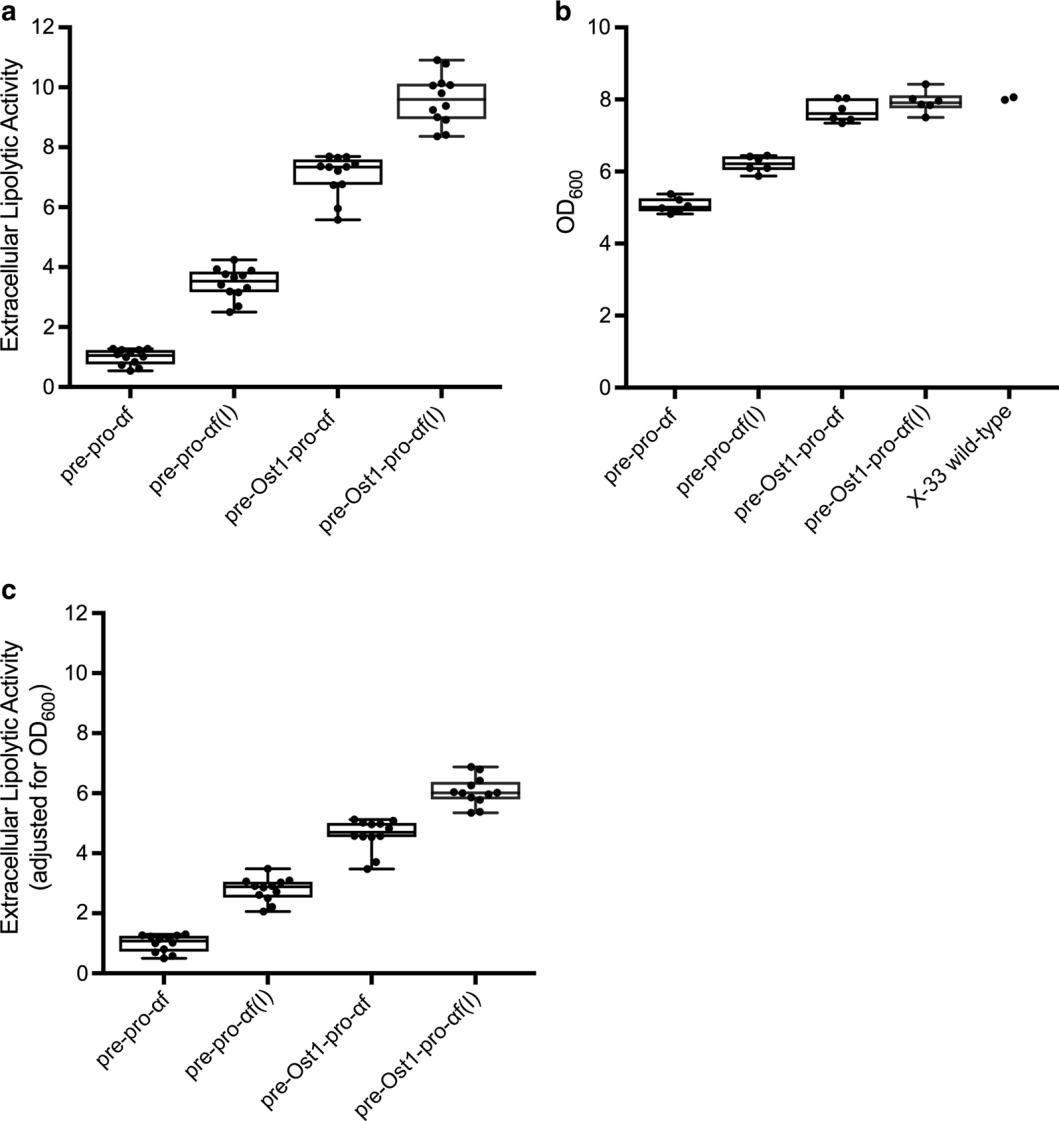
Extracellular BTL2 activity with the different secretion signals. This experiment was performed as in Fig. 2a, except that the secreted protein was BTL2. The abbreviations for the constructs are as in Fig. 2. **a** BTL2 lipolytic activity in the culture medium for the different secretion signals. The activity values were normalized by setting the value for the pre-pro-αf reference strain to 1. **b** Final OD_600_ values of the cultures at the end of the culture period. Two cultures of the parental X-33 wild-type strain were processed in parallel as a control. **c** Same as **a**, except that each lipolytic activity value was divided by the OD_600_ at the end of the incubation. The values were normalized by setting the value for the pre-pro-αf reference strain to 1

Is the enhanced secretion that we have documented for fluorescent proteins also seen for proteins of industrial interest? As a test case, we chose the BTL2 lipase, for the reasons outlined in the Introduction. Figure 6 shows the effects of different secretion signals on the secretion of BTL2 from *P. pastoris*, as determined by measuring lipolytic activity in the medium. The results are similar to those obtained with E2-Crimson. For constructs with the Ost1 signal sequence, lipolytic activity in the medium was considerably higher than for constructs with the α-factor signal sequence. For constructs with either the α-factor or Ost1 signal sequence, pro-α-factor(I) produced better results than pro-α-factor. These two effects were additive, and the improved secretion signal yielded about 10-fold more lipolytic activity in the medium than the original pre-pro-α-factor secretion signal (Fig. 6a).

One difference compared to the results with E2-Crimson was that the BTL2 constructs containing the α-factor signal sequence inhibited cell growth. At the end of the screening period, the OD_600_ of the cultures was about 20–40% lower for the constructs containing the α-factor signal sequence than for the cultures containing the Ost1 signal sequence (Fig. 6b). These variations in cell density account for some of the observed differences in secreted BTL2 levels. However, when the lipolytic activity in the medium was normalized to the OD_600_ of the cultures, the beneficial effects of the Ost1 signal sequence and pro-α-factor(I) were still clearly evident (Fig. 6c).

**Fig. 7:**
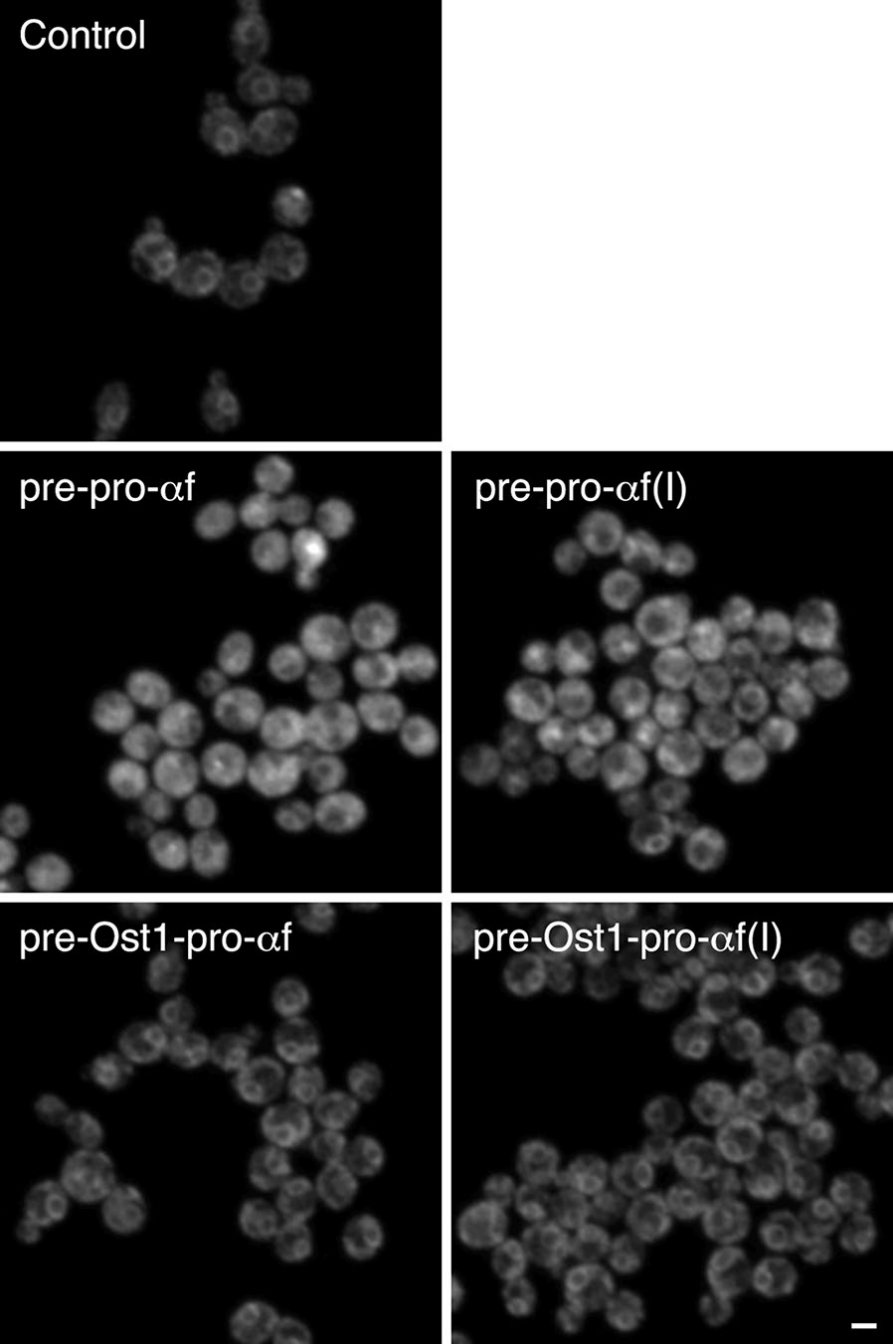
Clogging of ER translocons by posttranslational translocation of BTL2. This experiment was performed as in Fig. 4b, except that the secreted protein was BTL2. The abbreviations for the constructs are as in Fig. 2. Fluorescence images of ER-targeted GFP-HDEL are shown. In the “Control” sample, no BTL2 construct was expressed, and GFP-HDEL exhibited a typical ER pattern. For the BTL2 constructs with the α-factor signal sequence, most of the GFP-HDEL remained in the cytosol. For the BTL2 constructs with the Ost1 signal sequence, GFP-HDEL exhibited a typical ER pattern. Scale bar, 2 μm

Our interpretation is that like E2-Crimson, BTL2 can fold prematurely in the cytosol and clog the translocons during posttranslational translocation. To test this hypothesis, the strains expressing the BTL2 constructs were engineered to express ER-targeted GFP-HDEL. For cells expressing BTL2 constructs with the α-factor signal sequence, most of the fluorescence was cytosolic, whereas for cells expressing BTL2 constructs with the Ost1 signal sequence, most of the fluorescence showed a typical ER pattern (Fig. 7). This result supports the idea that efficient secretion of BTL2 requires cotranslational translocation.

## Discussion

We built on our previous cell biology-based approach [23] to devise an improved secretion signal for producing heterologous proteins in *P. pastoris*. The primary model protein was the fluorescent protein E2-Crimson, which was useful for three reasons. First, E2-Crimson can fold rapidly in the cytosol, so it serves as an example of a protein that requires efficient cotranslational translocation to enter the secretory pathway. Second, E2-Crimson is tetrameric, so it is likely to amplify any aggregation tendency of a secretion signal. Third, E2-Crimson emits red fluorescence, so it enables the detection of intracellular protein by fluorescence microscopy and of extracellular protein by fluorimetry. As described below, the analysis of E2-Crimson illuminated key features of the secretion signal.

In *S. cerevisiae*, the N-terminal signal sequence plays a major role in determining whether translocation is cotranslational or posttranslational [21, 43, 44]. Most soluble proteins that enter the *S. cerevisiae* secretory pathway, including pre-pro-α-factor, undergo posttranslational translocation [45]. However, the Ost1 signal sequence directs efficient cotranslational translocation in *S. cerevisiae* [23, 24, 46]. Although translocation into the ER has not been studied in *P. pastoris*, the mechanisms are probably similar to those in *S. cerevisiae*. Indeed, we reported that replacement of the α-factor signal sequence with the Ost1 signal sequence strongly increased secretion of msGFP in *P. pastoris* [23]. A similar beneficial effect of the Ost1 signal sequence is described here for secretion of E2-Crimson in *P. pastoris*.

After reaching the ER lumen, proteins can be exported by bulk flow, but this process is relatively slow [47]. Faster export is mediated by signal-dependent ER export receptors that concentrate secretory proteins in COPII vesicles [9, 48]. For example, the α-factor pro region contains an ER export signal that is recognized by the transmembrane Erv29 receptor [9, 49, 50]. This receptor-driven export probably helps to explain why the α-factor secretion signal is often effective for heterologous protein production. Yet the presence of the α-factor pro region in a secretion signal also carries risk, because the pro region remains attached to the secretory protein until being removed by the Kex2 processing protease in the Golgi [10]. When a secretory protein is present at high levels in the ER lumen, the α-factor pro region might cause aggregation, particularly if the secretory protein is oligomeric. Such aggregation in the ER was seen when the α-factor pro region was linked to E2-Crimson. By contrast, an allelic variant of the α-factor pro region caused no aggregation of E2-Crimson. We traced this effect to amino acid 42 in the pro region, where Leu42 promotes aggregation but Ser42 does not. The Ser42 variant is present in the widely used *P.* *pastoris* expression vectors supplied by Invitrogen, and it has likely benefited *P.* *pastoris* researchers who used those vectors. Thus, α-factor pro region variants that contain Ser42 can be used to drive rapid signal-mediated ER exit of a heterologous protein without the side effect of promoting ER aggregation. The combined effect of the Ost1 signal sequence and a Ser42 variant of the α-factor pro region was dramatic—compared to the Leu42 variant of the α-factor secretion signal, the improved secretion signal enhanced secretion of E2-Crimson approximately 20-fold.

To test whether the benefits of the improved secretion signal extend to secreted proteins of practical value, we tested the lipase BTL2. This protein was a good candidate for four reasons. First, *P. pastoris* had previously been engineered to secrete active BTL2 [33]. Second, even though BTL2 is naturally produced as an extracellular enzyme [30], it was active when expressed intracellularly in *E. coli* [32], indicating that the protein can fold in the cytosol. Third, the secretion of BTL2 from *S. cerevisiae* was recently found to be influenced by the signal sequence [34]. Fourth, although BTL2 is a monomeric protein, it tends to aggregate at high concentrations [29, 51]. These properties suggested that BLT2 would benefit from cotranslational translocation into the ER and from the presence of Ser42 in the α-factor pro region. Indeed, both the Ost1 signal sequence and a Ser42 variant of the α-factor pro region enhanced secretion of BTL2. Compared to the Leu42 variant of the α-factor secretion signal, the improved secretion signal resulted in better growth of the BTL2-expressing cells, and enhanced the yield of secreted BTL2 approximately 10-fold.

As part of this analysis, we developed a method to assess whether a heterologous protein fused to the α-factor secretion signal accumulates in the *P. pastoris* cytosol and clogs the ER translocons. The method employs a *P. pastoris* strain that expresses an ER-targeted GFP-HDEL construct, which normally gives a distinctive fluorescence pattern. When the translocons are clogged due to failed posttranslational translocation, GFP fluorescence shifts to the cytosol. Both E2-Crimson and BTL2 produced such a shift in GFP fluorescence. For the future, we plan to test whether this assay reliably identifies heterologous proteins that will be secreted more efficiently with the improved secretion signal.

## Conclusions

By combining the Ost1 signal sequence with a Ser42 variant of the α-factor pro region, we obtained an improved secretion signal for *P. pastoris*. With the E2-Crimson and BTL2 model proteins, this secretion signal boosted secretion up to 20- and 10-fold, respectively, relative to the Leu42 variant of the α-factor secretion signal. It will be interesting to test whether the improved secretion signal enhances the secretion from *P. pastoris* of other proteins, particularly proteins that can fold prior to translocation and proteins that oligomerize in the ER, and whether these enhancements are also seen at high cell densities in bioreactor-scale fermentations. If so, the improved secretion signal could replace the common variants of the α-factor secretion signal as the default standard for producing heterologous proteins in *P. pastoris*.

## Additional file

**Additional file 1: Figure S1.** The *S. cerevisiae* α-factor secretion signal. Depicted is the 5′ portion of the *MFα1* gene encoding pre-pro-α-factor. This sequence encodes the Leu42 variant. Cleavage sites for signal peptidase and Kex2 are marked, and Leu42 and Asp83 are highlighted. This image was generated using SnapGene software. **Figure S2.** Low variation in secreted protein levels between clones. Each of the indicated constructs was transformed into *P. pastoris* cells, and six independent clones with confirmed single integrations were cultured and then analyzed as in Fig. 2a to measure extracellular protein. For each construct, the average protein level for the six clones was defined as 1.0. Two representative clones of a given construct were chosen for further analysis. The red boxes represent E2-Crimson constructs, and the black boxes represent BTL2 constructs. **Figure S3.** Immunoblot showing cell-associated and secreted E2-Crimson. Strains that expressed E2-Crimson fused to the indicated secretion signals were grown and induced. Fractions containing cell-associated E2-Crimson (“C”) or E2-Crimson in the extracellular medium (“M”) were subjected to SDS-PAGE and immunoblotting. The molecular weight (“MW”) marker was the Precision Plus Protein Dual Color Standards (Bio-Rad). Molecular weights of the protein standards are indicated. **Figure S4.** Large image fields of cells expressing the various constructs. From the experiment of Fig. 3, large image fields were captured to illustrate that the fluorescence patterns were consistent among the cells in a population, and that cells expressing constructs with the α-factor signal sequence were often unusually large. Scale bar, 2 μm. **Figure S5.** Distribution of ER-targeted GFP-HDEL before induced expression of E2-Crimson constructs. The same cultures shown in Fig. 4b were examined by fluorescence microscopy before methanol-induced expression of the E2-Crimson constructs. All of the strains showed a typical ER pattern for GFP-HDEL. Scale bar, 2 μm. **Figure S6.** Theoretical prediction of aggregation propensities in the two allelic variants of the α-factor pro region. Left, the Leu42 variant of the α-factor pro region was analyzed using the online AGGRESCAN tool (http://bioinf.uab.es/aap/). The position of Leu42 in a predicted aggregation-prone region is marked. Right, the same analysis was performed for the Ser42 variant of the α-factor pro region.

**Additional file 2:** Compressed folder of SnapGene files for the constructs used in this study. These files can be opened with the free SnapGene Viewer (http://www.snapgene.com/products/snapgene_viewer/).

## Abbreviations

BMG: buffered minimal glycerol medium
BMM: buffered minimal methanol medium
ER: endoplasmic reticulum
msGFP: monomeric superfolder GFP
OD_600_: optical density at 600 nm
PBS: phosphate buffered saline
YPD: rich yeast medium

## References

[1] Corchero JL, Gasser B, Resina D, Smith W, Parrilli E, Vázquez F (2013). Unconventional microbial systems for the cost-efficient production of high-quality protein therapeutics. Biotechnol Adv.

[2] Tran AM, Nguyen TT, Nguyen CT, Huynh-Thi XM, Nguyen CT, Trinh MT (2017). *Pichia pastoris* versus *Saccharomyces cerevisiae*: a case study on the recombinant production of human granulocyte-macrophage colony-stimulating factor. BMC Res Notes.

[3] Darby RAJ, Cartwright SP, Dilworth MV, Bill RM (2012). Which yeast species shall I choose? *Saccharomyces cerevisiae* versus *Pichia pastoris* (Review).. Recomb Protein Prod Yeast Methods Protoc..

[4] Ahmad M, Hirz M, Pichler H, Schwab H (2014). Protein expression in *Pichia pastoris*: recent achievements and perspectives for heterologous protein production. Appl Microbiol Biotechnol.

[5] Shay LK, Hunt HR, Wegner GH (1987). High-productivity fermentation process for cultivating industrial microorganisms. J Ind Microbiol.

[6] Cregg JM, Madden KR, Barringer KJ, Thill GP, Stillman CA (1989). Functional characterization of the two alcohol oxidase genes from the yeast *Pichia pastoris*. Mol Cell Biol.

[7] Brake AJ, Merryweather JP, Coit DG, Heberlein UA, Masiarz FR, Mullenbach GT (1984). Alpha-factor-directed synthesis and secretion of mature foreign proteins in *Saccharomyces cerevisiae*. Proc Natl Acad Sci USA.

[8] Otte S, Barlowe C (2004). Sorting signals can direct receptor-mediated export of soluble proteins into COPII vesicles. Nat Cell Biol.

[9] Dancourt J, Barlowe C (2010). Protein sorting receptors in the early secretory pathway. Annu Rev Biochem.

[10] Fuller RS, Sterne RE, Thorner J (1988). Enzymes required for yeast prohormone processing. Annu Rev Physiol.

[11] Julius D, Blair L, Brake A, Sprague G, Thorner J (1983). Yeast α factor is processed from a larger precursor polypeptide: the essential role of a membrane-bound dipeptidyl aminopeptidase. Cell.

[12] Puxbaum V, Mattanovich D, Gasser B (2015). Quo vadis? The challenges of recombinant protein folding and secretion in *Pichia pastoris*. Appl Microbiol Biotechnol.

[13] Looser V, Bruhlmann B, Bumbak F, Stenger C, Costa M, Camattari A (2015). Cultivation strategies to enhance productivity of *Pichia pastoris*: a review. Biotechnol Adv.

[14] Gasser B, Prielhofer R, Marx H, Maurer M, Nocon J, Steiger M (2013). *Pichia pastoris*: protein production host and model organism for biomedical research. Future Microbiol..

[15] Sturmberger L, Chappell T, Geier M, Krainer F, Day KJ, Vide U (2016). Refined *Pichia pastoris* reference genome sequence. J Biotechnol.

[16] Daly R, Hearn MTW (2005). Expression of heterologous proteins in *Pichia pastoris*: a useful experimental tool in protein engineering and production. J Mol Recognit.

[17] Lin-Cereghino GP, Stark CM, Kim D, Chang J, Shaheen N, Poerwanto H (2013). The effect of α-mating factor secretion signal mutations on recombinant protein expression in *Pichia pastoris*. Gene.

[18] Joo HH, Xue L, Tsai JW, Park SPJ, Kwon J, Patel A (2017). Structural characterization of the α-mating factor prepro-peptide for secretion of recombinant proteins in *Pichia pastoris*. Gene.

[19] Massahi A, Çalik P (2015). In-silico determination of Pichia pastoris signal peptides for extracellular recombinant protein production. J Theor Biol.

[20] Ahn J, Jang MJ, Ang KS, Lee H, Choi ES, Lee DY (2016). Codon optimization of Saccharomyces cerevisiae mating factor alpha prepro-leader to improve recombinant protein production in Pichia pastoris. Biotechnol Lett.

[21] Ng DTW, Brown JD, Walter P (1996). Signal sequences specify the targeting route to the endoplasmic reticulum membrane. J Cell Biol.

[22] Plath K, Mothes W, Wilkinson BM, Stirling CJ, Rapoport TA (1998). Signal sequence recognition in posttranslational protein transport across the yeast ER membrane. Cell.

[23] Fitzgerald I, Glick BS (2014). Secretion of a foreign protein from budding yeasts is enhanced by cotranslational translocation and by suppression of vacuolar targeting. Microb Cell Fact.

[24] Willer M, Forte GMA, Stirling CJ (2008). Sec61p is required for ERAD-L: genetic dissection of the translocation and ERAD-L functions of Sec61P using novel derivatives of CPY. J Biol Chem.

[25] Forte GMA, Pool MR, Stirling CJ (2011). N-terminal acetylation inhibits protein targeting to the endoplasmic reticulum. PLoS Biol..

[26] Strack RL, Hein B, Bhattacharyya D, Hell SW, Keenan RJ, Glick BS (2009). A rapidly maturing far-red derivative of DsRed-Express2 for whole-cell labeling. Biochemistry.

[27] Surribas A, Resina D, Ferrer P, Valero F (2007). Rivoflavin may interfere with on-line monitoring of secreted green fluorescence protein fusion proteins in *Pichia pastoris*. Microb Cell Fact.

[28] Marx H, Mattanovich D, Sauer M (2008). Overexpression of the riboflavin biosynthetic pathway in *Pichia pastoris*. Microb Cell Fact.

[29] Rúa ML, Schmidt-Dannert C, Wahl S, Sprauer A, Schmid RD (1997). Thermoalkalophilic lipase of *Bacillus thermocatenulatus*: arge-scale production, purification and properties: aggregation behaviour and its effect on activity. J Biotechnol..

[30] Schmidt-Dannert C, Rúa ML, Atomi H, Schmid RD (1996). Thermoalkalophilic lipase of *Bacillus thermocatenulatus*. I. Molecular cloning, nucleotide sequence, purification and some properties. Biochim Biophys Acta.

[31] Hasan F, Shah AA, Hameed A (2009). Methods for detection and characterization of lipases: a comprehensive review. Biotechnol Adv.

[32] Rúa ML, Atomi H, Schmidt-Dannert C, Schmid RD (1998). High-level expression of the thermoalkalophilic lipase from Bacillus thermocatenulatus in *Escherichia coli*. Appl Microbiol Biotechnol.

[33] Quyen DT, Schmidt-Dannert C, Schmid RD (2003). High-level expression of a lipase from Bacillus thermocatenulatus BTL2 in *Pichia pastoris* and some properties of the recombinant lipase. Protein Expr Purif.

[34] Kajiwara S, Yamada R, Ogino H (2018). Secretory overexpression of *Bacillus thermocatenulatus* lipase in *Saccharomyces cerevisiae* using combinatorial library strategy. Biotechnol J.

[35] Wenyan Wang BAM (1999). Two-stage PCR protocol allowing introduction of multiple mutations, deletions and insertions using QuikChange site-directed mutagenesis. Biotechniques.

[36] Sears IB, O’Connor J, Rossanese OW, Glick BS (1998). A versatile set of vectors for constitutive and regulated gene expression in *Pichia pastoris*. Yeast.

[37] Cámara E, Albiol J, Ferrer P (2016). Droplet digital PCR-aided screening and characterization of *Pichia pastoris* multiple gene copy strains. Biotechnol Bioeng.

[38] Rakestraw JA, Sazinsky SL, Piatesi A, Antipov E, Wittrup KD (2009). Directed evolution of a secretory leader for the improved expression of heterologous proteins and full-length antibodies in *Saccharomyces cerevisiae*. Biotechnol Bioeng.

[39] Kurjan J, Herskowitz I (1982). Structure of a yeast pheromone gene (MFα): a putative α-factor precursor contains four tandem copies of mature α-factor. Cell.

[40] Ast T, Michaelis S, Schuldiner M, States U (2017). The protease Ste24 clears clogged translocons. Cell.

[41] Rossanese OW, Reinke CA, Bevis BJ, Hammond AT, Sears IB, O’Connor J (2001). A role for actin, Cdc1p, and Myo2p in the inheritance of late Golgi elements in *Saccharomyces cerevisiae*. J Cell Biol.

[42] Conchillo-Solé O, de Groot NS, Avilés FX, Vendrell J, Daura X, Ventura S (2007). AGGRESCAN: a server for the prediction and evaluation of “hot spots” of aggregation in polypeptides. BMC Bioinform.

[43] Mandon EC, Trueman SF, Gilmore R (2013). Protein translocation across the rough endoplasmic reticulum. Cold Spring Harb Perspect Biol.

[44] Benham AM (2012). Protein secretion and the endoplasmic reticulum. Cold Spring Harb Perspect Biol.

[45] Ast T, Cohen G, Schuldiner M (2013). A network of cytosolic factors targets SRP-independent proteins to the endoplasmic reticulum. Cell.

[46] Pechmann S, Chartron JW, Frydman J (2014). Local slowdown of translation by nonoptimal codons promotes nascent-chain recognition by SRP in vivo. Nat Struct Mol Biol.

[47] Barlowe C, Helenius A (2016). Cargo capture and bulk flow in the early secretory pathway. Annu Rev Cell Dev Biol.

[48] Barlowe CK, Miller EA (2013). Secretory protein biogenesis and traffic in the early secretory pathway. Genetics.

[49] Foley DA, Sharpe HJ, Otte S (2007). Membrane topology of the endoplasmic reticulum to Golgi transport factor Erv29p. Mol Membr Biol.

[50] Belden WJ, Barlowe C (2001). Role of Erv29p in collecting soluble secretory proteins into ER-derived transport vesicles. Science.

[51] Schlieben NH, Niefind K, Schomburg D (2004). Expression, purification, and aggregation studies of His-tagged thermoalkalophilic lipase from *Bacillus thermocatenulatus*. Protein Expr Purif.

