# Supplementary figures and images for: An improved secretion signal enhances the secretion of model proteins from Pichia pastoris
Source: Microb Cell Fact. 2018 Oct 12;17:161. doi: 10.1186/s12934-018-1009-5 (PMC6182871; doi:10.1186/s12934-018-1009-5)

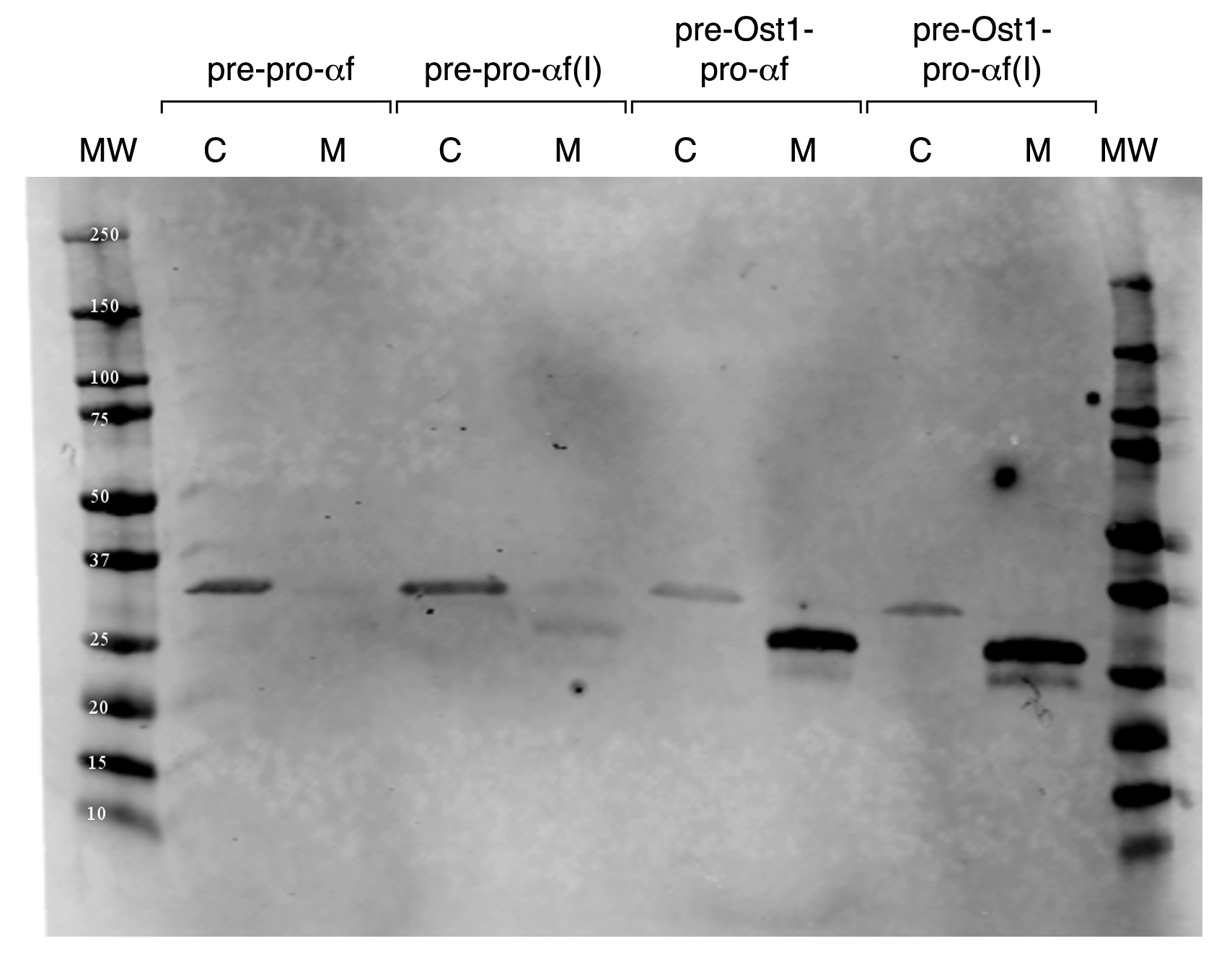

Supplement: Supplementary file 1 — Additional file 1: Figure S1. The S. cerevisiae α-factor secretion signal. Depicted is the 5′ portion of the MFα1 gene encoding pre-pro-α-factor. This sequence encodes the Leu42 variant. Cleavage sites for signal peptidase and Kex2 are marked, and Leu42 and Asp83 are highlighted. This image was generated using SnapGene software. Figure S2. Low variation in secreted protein levels between clones. Each of the indicated constructs was transformed into P. pastoris cells, and six independent clones with confirmed single integrations were cultured and then analyzed as in Fig. 2a to measure extracellular protein. For each construct, the average protein level for the six clones was defined as 1.0. Two representative clones of a given construct were chosen for further analysis. The red boxes represent E2-Crimson constructs, and the black boxes represent BTL2 constructs. Figure S3. Immunoblot showing cell-associated and secreted E2-Crimson. Strains that expressed E2-Crimson fused to the indicated secretion signals were grown and induced. Fractions containing cell-associated E2-Crimson (“C”) or E2-Crimson in the extracellular medium (“M”) were subjected to SDS-PAGE and immunoblotting. The molecular weight (“MW”) marker was the Precision Plus Protein Dual Color Standards (Bio-Rad). Molecular weights of the protein standards are indicated. Figure S4. Large image fields of cells expressing the various constructs. From the experiment of Fig. 3, large image fields were captured to illustrate that the fluorescence patterns were consistent among the cells in a population, and that cells expressing constructs with the α-factor signal sequence were often unusually large. Scale bar, 2 μm. Figure S5. Distribution of ER-targeted GFP-HDEL before induced expression of E2-Crimson constructs. The same cultures shown in Fig. 4b were examined by fluorescence microscopy before methanol-induced expression of the E2-Crimson constructs. All of the strains showed a typical ER pattern for GFP-HDEL. S [file 12934_2018_1009_MOESM1_ESM.zip › Figure S3.tif]

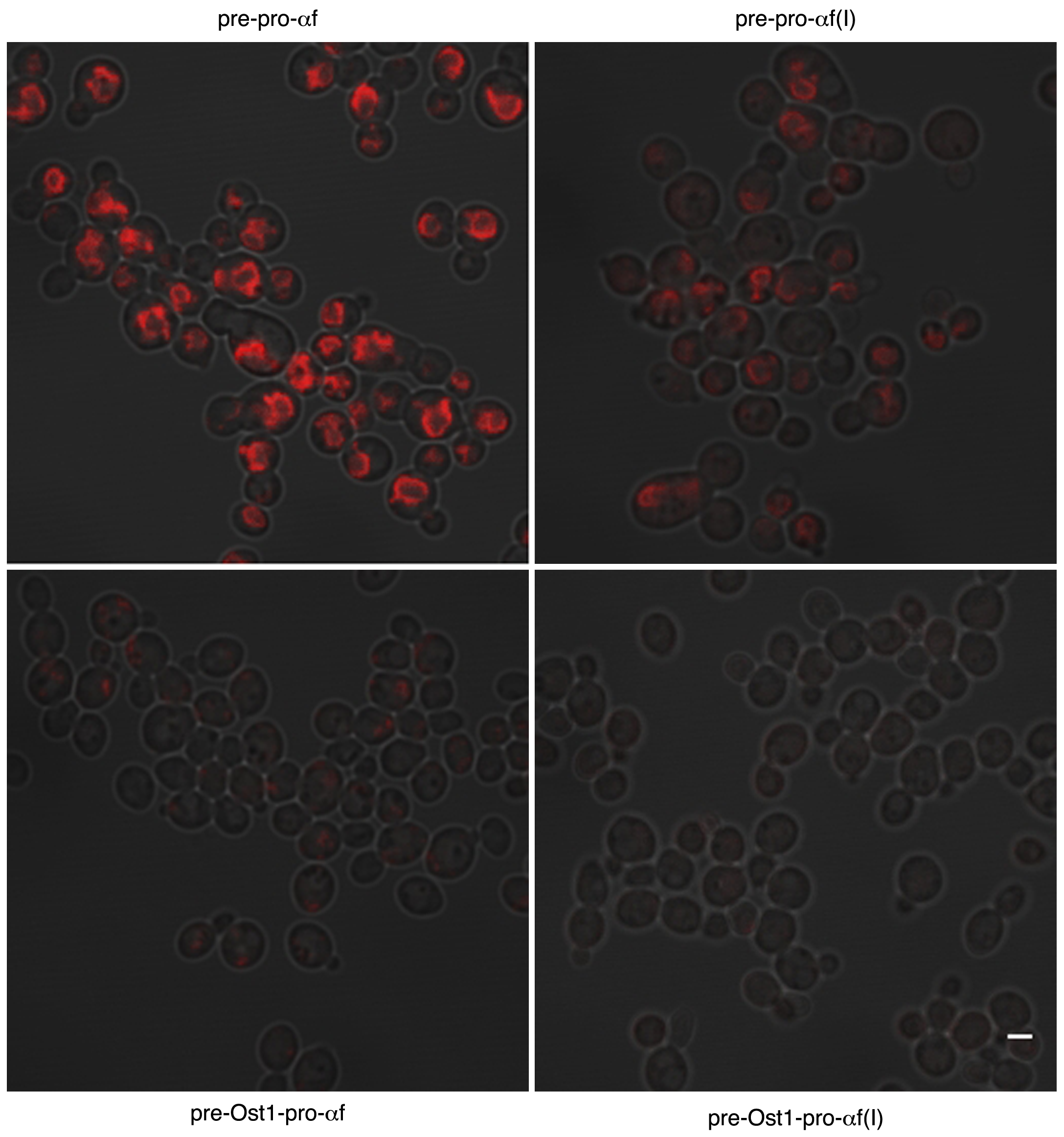

Supplement: Supplementary file 1 — Additional file 1: Figure S1. The S. cerevisiae α-factor secretion signal. Depicted is the 5′ portion of the MFα1 gene encoding pre-pro-α-factor. This sequence encodes the Leu42 variant. Cleavage sites for signal peptidase and Kex2 are marked, and Leu42 and Asp83 are highlighted. This image was generated using SnapGene software. Figure S2. Low variation in secreted protein levels between clones. Each of the indicated constructs was transformed into P. pastoris cells, and six independent clones with confirmed single integrations were cultured and then analyzed as in Fig. 2a to measure extracellular protein. For each construct, the average protein level for the six clones was defined as 1.0. Two representative clones of a given construct were chosen for further analysis. The red boxes represent E2-Crimson constructs, and the black boxes represent BTL2 constructs. Figure S3. Immunoblot showing cell-associated and secreted E2-Crimson. Strains that expressed E2-Crimson fused to the indicated secretion signals were grown and induced. Fractions containing cell-associated E2-Crimson (“C”) or E2-Crimson in the extracellular medium (“M”) were subjected to SDS-PAGE and immunoblotting. The molecular weight (“MW”) marker was the Precision Plus Protein Dual Color Standards (Bio-Rad). Molecular weights of the protein standards are indicated. Figure S4. Large image fields of cells expressing the various constructs. From the experiment of Fig. 3, large image fields were captured to illustrate that the fluorescence patterns were consistent among the cells in a population, and that cells expressing constructs with the α-factor signal sequence were often unusually large. Scale bar, 2 μm. Figure S5. Distribution of ER-targeted GFP-HDEL before induced expression of E2-Crimson constructs. The same cultures shown in Fig. 4b were examined by fluorescence microscopy before methanol-induced expression of the E2-Crimson constructs. All of the strains showed a typical ER pattern for GFP-HDEL. S [file 12934_2018_1009_MOESM1_ESM.zip › Figure S4.tif]

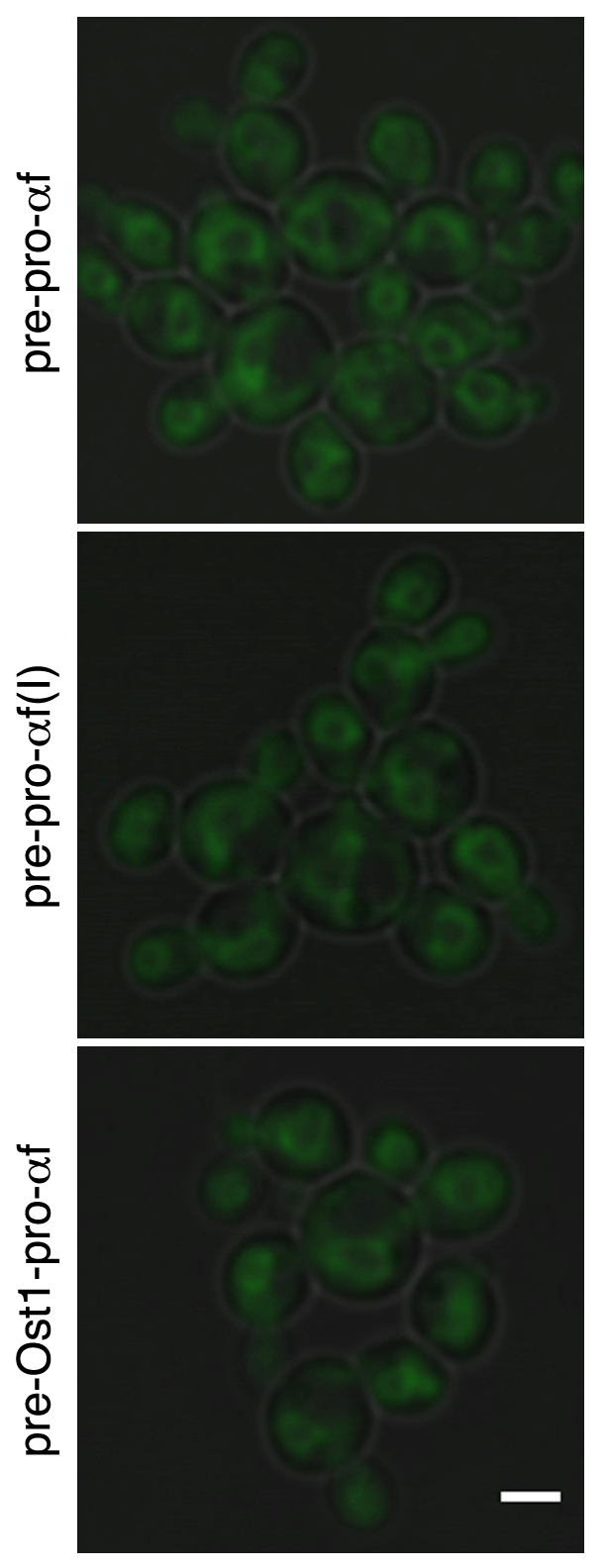

Supplement: Supplementary file 1 — Additional file 1: Figure S1. The S. cerevisiae α-factor secretion signal. Depicted is the 5′ portion of the MFα1 gene encoding pre-pro-α-factor. This sequence encodes the Leu42 variant. Cleavage sites for signal peptidase and Kex2 are marked, and Leu42 and Asp83 are highlighted. This image was generated using SnapGene software. Figure S2. Low variation in secreted protein levels between clones. Each of the indicated constructs was transformed into P. pastoris cells, and six independent clones with confirmed single integrations were cultured and then analyzed as in Fig. 2a to measure extracellular protein. For each construct, the average protein level for the six clones was defined as 1.0. Two representative clones of a given construct were chosen for further analysis. The red boxes represent E2-Crimson constructs, and the black boxes represent BTL2 constructs. Figure S3. Immunoblot showing cell-associated and secreted E2-Crimson. Strains that expressed E2-Crimson fused to the indicated secretion signals were grown and induced. Fractions containing cell-associated E2-Crimson (“C”) or E2-Crimson in the extracellular medium (“M”) were subjected to SDS-PAGE and immunoblotting. The molecular weight (“MW”) marker was the Precision Plus Protein Dual Color Standards (Bio-Rad). Molecular weights of the protein standards are indicated. Figure S4. Large image fields of cells expressing the various constructs. From the experiment of Fig. 3, large image fields were captured to illustrate that the fluorescence patterns were consistent among the cells in a population, and that cells expressing constructs with the α-factor signal sequence were often unusually large. Scale bar, 2 μm. Figure S5. Distribution of ER-targeted GFP-HDEL before induced expression of E2-Crimson constructs. The same cultures shown in Fig. 4b were examined by fluorescence microscopy before methanol-induced expression of the E2-Crimson constructs. All of the strains showed a typical ER pattern for GFP-HDEL. S [file 12934_2018_1009_MOESM1_ESM.zip › Figure S5.tif]

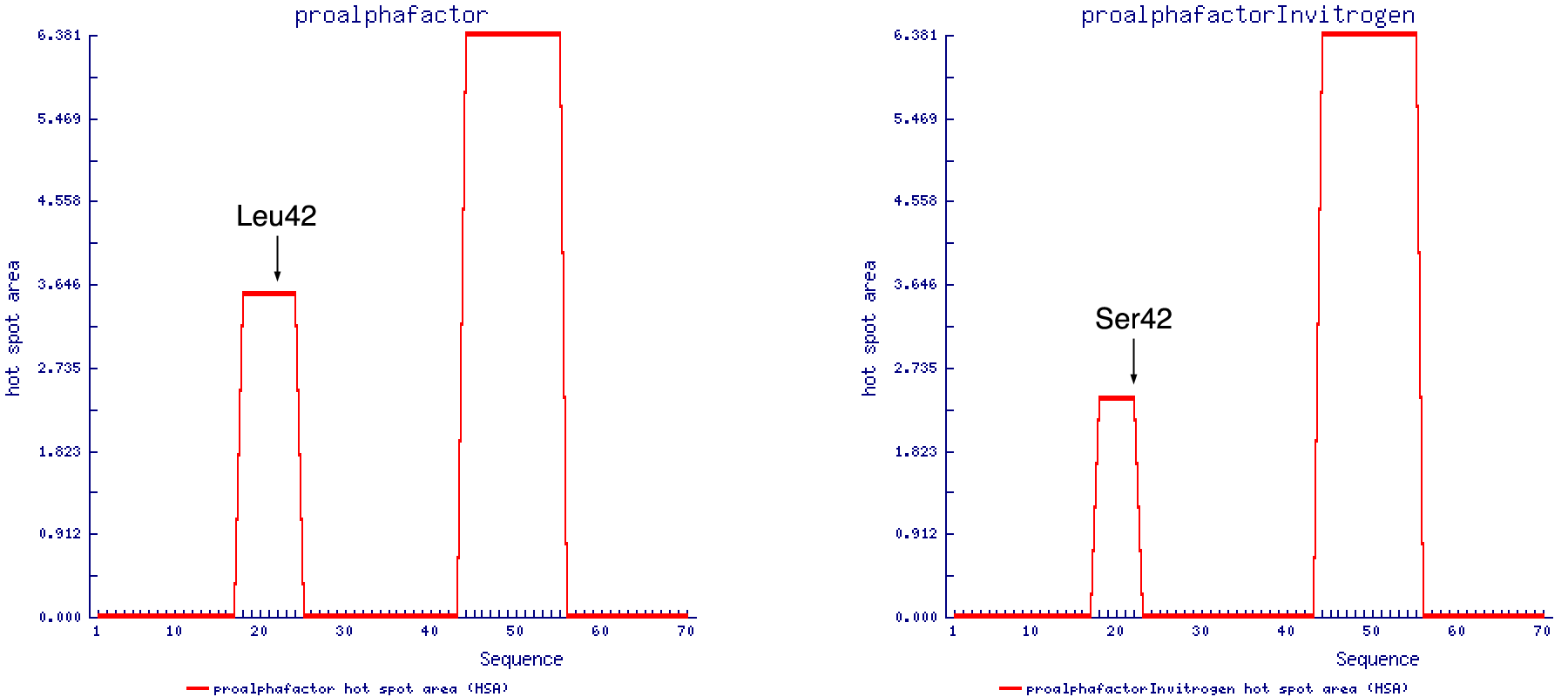

Supplement: Supplementary file 1 — Additional file 1: Figure S1. The S. cerevisiae α-factor secretion signal. Depicted is the 5′ portion of the MFα1 gene encoding pre-pro-α-factor. This sequence encodes the Leu42 variant. Cleavage sites for signal peptidase and Kex2 are marked, and Leu42 and Asp83 are highlighted. This image was generated using SnapGene software. Figure S2. Low variation in secreted protein levels between clones. Each of the indicated constructs was transformed into P. pastoris cells, and six independent clones with confirmed single integrations were cultured and then analyzed as in Fig. 2a to measure extracellular protein. For each construct, the average protein level for the six clones was defined as 1.0. Two representative clones of a given construct were chosen for further analysis. The red boxes represent E2-Crimson constructs, and the black boxes represent BTL2 constructs. Figure S3. Immunoblot showing cell-associated and secreted E2-Crimson. Strains that expressed E2-Crimson fused to the indicated secretion signals were grown and induced. Fractions containing cell-associated E2-Crimson (“C”) or E2-Crimson in the extracellular medium (“M”) were subjected to SDS-PAGE and immunoblotting. The molecular weight (“MW”) marker was the Precision Plus Protein Dual Color Standards (Bio-Rad). Molecular weights of the protein standards are indicated. Figure S4. Large image fields of cells expressing the various constructs. From the experiment of Fig. 3, large image fields were captured to illustrate that the fluorescence patterns were consistent among the cells in a population, and that cells expressing constructs with the α-factor signal sequence were often unusually large. Scale bar, 2 μm. Figure S5. Distribution of ER-targeted GFP-HDEL before induced expression of E2-Crimson constructs. The same cultures shown in Fig. 4b were examined by fluorescence microscopy before methanol-induced expression of the E2-Crimson constructs. All of the strains showed a typical ER pattern for GFP-HDEL. S [file 12934_2018_1009_MOESM1_ESM.zip › Figure S6.tif]

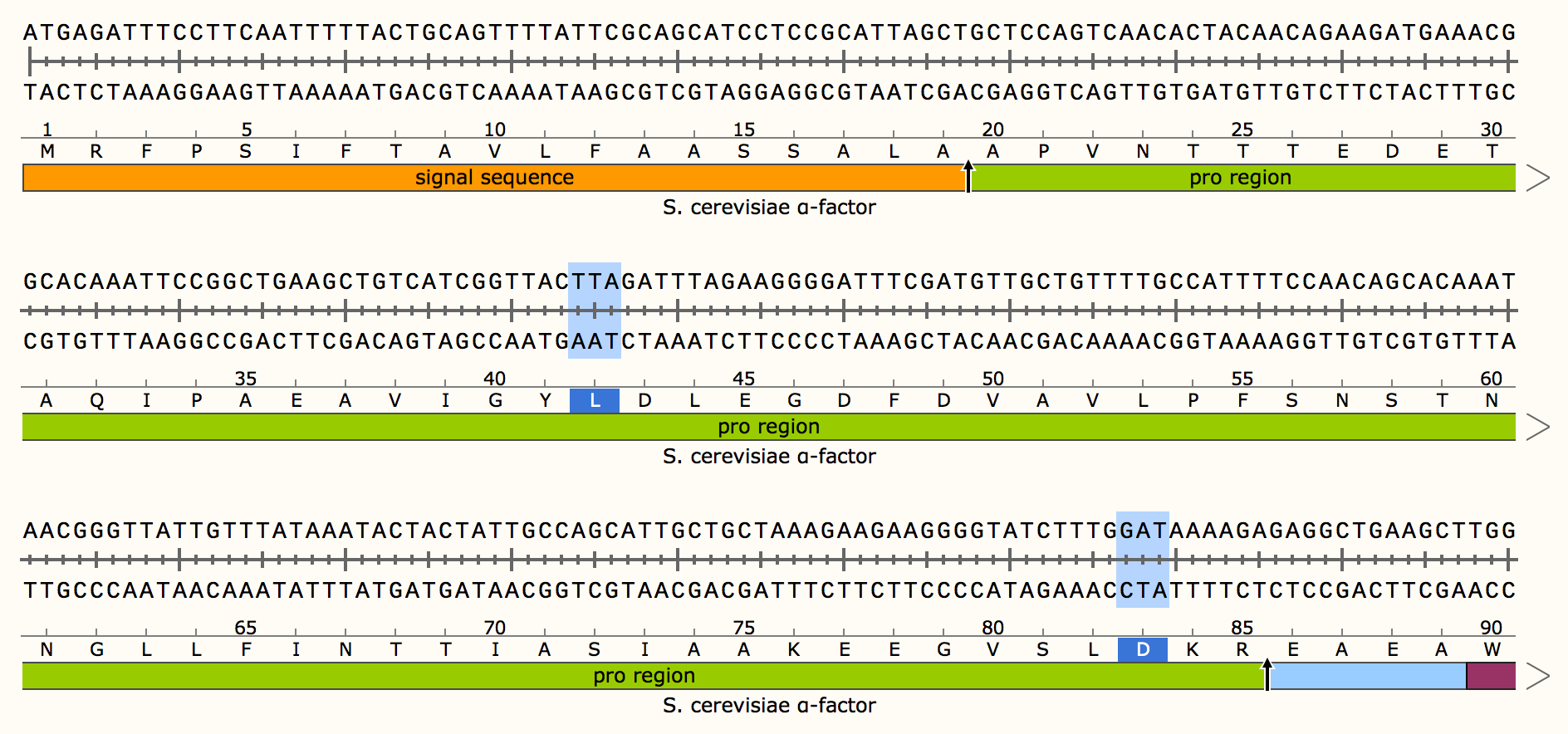

Supplement: Supplementary file 1 — Additional file 1: Figure S1. The S. cerevisiae α-factor secretion signal. Depicted is the 5′ portion of the MFα1 gene encoding pre-pro-α-factor. This sequence encodes the Leu42 variant. Cleavage sites for signal peptidase and Kex2 are marked, and Leu42 and Asp83 are highlighted. This image was generated using SnapGene software. Figure S2. Low variation in secreted protein levels between clones. Each of the indicated constructs was transformed into P. pastoris cells, and six independent clones with confirmed single integrations were cultured and then analyzed as in Fig. 2a to measure extracellular protein. For each construct, the average protein level for the six clones was defined as 1.0. Two representative clones of a given construct were chosen for further analysis. The red boxes represent E2-Crimson constructs, and the black boxes represent BTL2 constructs. Figure S3. Immunoblot showing cell-associated and secreted E2-Crimson. Strains that expressed E2-Crimson fused to the indicated secretion signals were grown and induced. Fractions containing cell-associated E2-Crimson (“C”) or E2-Crimson in the extracellular medium (“M”) were subjected to SDS-PAGE and immunoblotting. The molecular weight (“MW”) marker was the Precision Plus Protein Dual Color Standards (Bio-Rad). Molecular weights of the protein standards are indicated. Figure S4. Large image fields of cells expressing the various constructs. From the experiment of Fig. 3, large image fields were captured to illustrate that the fluorescence patterns were consistent among the cells in a population, and that cells expressing constructs with the α-factor signal sequence were often unusually large. Scale bar, 2 μm. Figure S5. Distribution of ER-targeted GFP-HDEL before induced expression of E2-Crimson constructs. The same cultures shown in Fig. 4b were examined by fluorescence microscopy before methanol-induced expression of the E2-Crimson constructs. All of the strains showed a typical ER pattern for GFP-HDEL. S [file 12934_2018_1009_MOESM1_ESM.zip › Figure S1.tif]

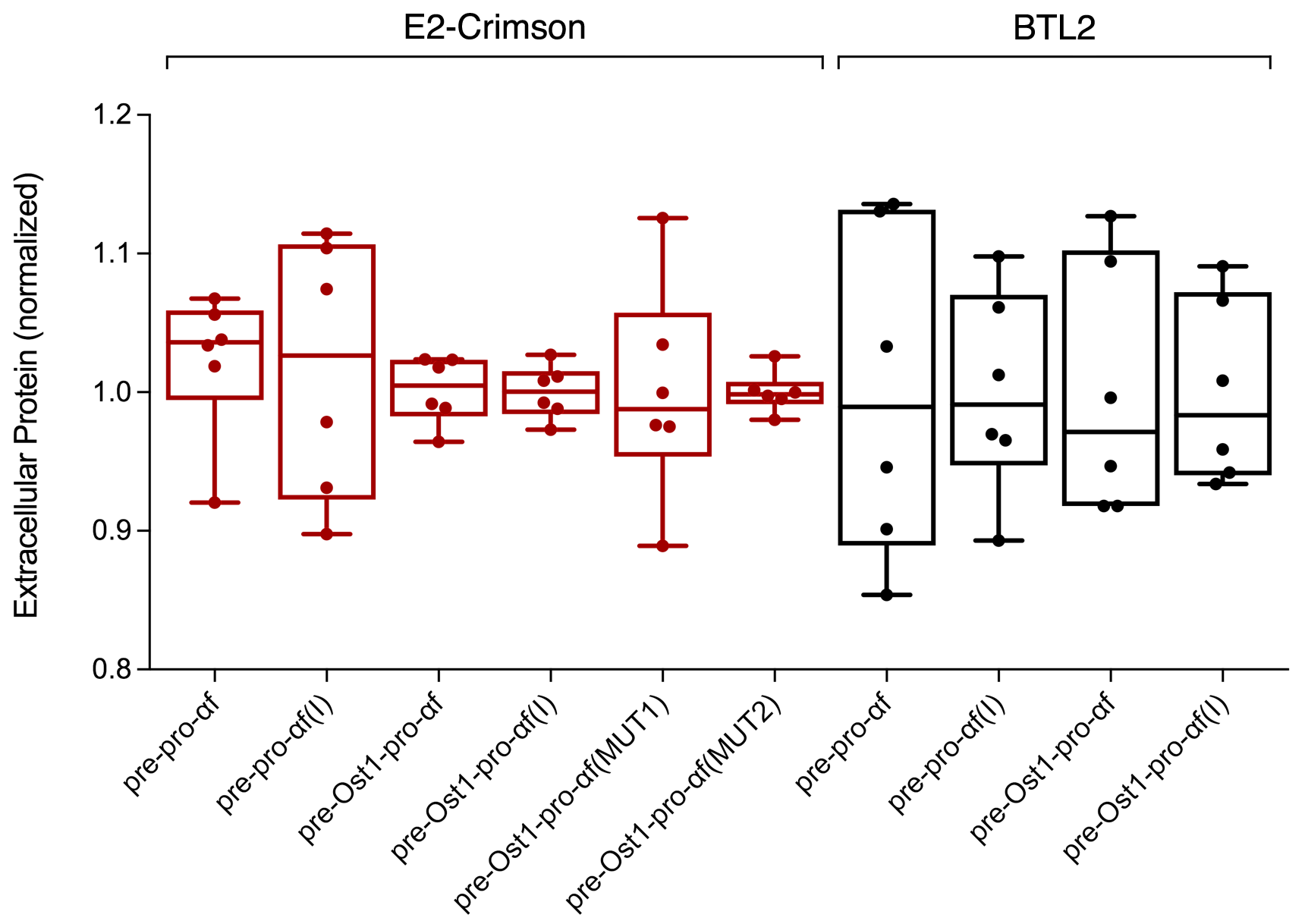

Supplement: Supplementary file 1 — Additional file 1: Figure S1. The S. cerevisiae α-factor secretion signal. Depicted is the 5′ portion of the MFα1 gene encoding pre-pro-α-factor. This sequence encodes the Leu42 variant. Cleavage sites for signal peptidase and Kex2 are marked, and Leu42 and Asp83 are highlighted. This image was generated using SnapGene software. Figure S2. Low variation in secreted protein levels between clones. Each of the indicated constructs was transformed into P. pastoris cells, and six independent clones with confirmed single integrations were cultured and then analyzed as in Fig. 2a to measure extracellular protein. For each construct, the average protein level for the six clones was defined as 1.0. Two representative clones of a given construct were chosen for further analysis. The red boxes represent E2-Crimson constructs, and the black boxes represent BTL2 constructs. Figure S3. Immunoblot showing cell-associated and secreted E2-Crimson. Strains that expressed E2-Crimson fused to the indicated secretion signals were grown and induced. Fractions containing cell-associated E2-Crimson (“C”) or E2-Crimson in the extracellular medium (“M”) were subjected to SDS-PAGE and immunoblotting. The molecular weight (“MW”) marker was the Precision Plus Protein Dual Color Standards (Bio-Rad). Molecular weights of the protein standards are indicated. Figure S4. Large image fields of cells expressing the various constructs. From the experiment of Fig. 3, large image fields were captured to illustrate that the fluorescence patterns were consistent among the cells in a population, and that cells expressing constructs with the α-factor signal sequence were often unusually large. Scale bar, 2 μm. Figure S5. Distribution of ER-targeted GFP-HDEL before induced expression of E2-Crimson constructs. The same cultures shown in Fig. 4b were examined by fluorescence microscopy before methanol-induced expression of the E2-Crimson constructs. All of the strains showed a typical ER pattern for GFP-HDEL. S [file 12934_2018_1009_MOESM1_ESM.zip › Figure S2.tif]
